# *Post-hoc* analysis of a randomized controlled trial on the impact of pre-transplant use of probiotics on outcomes after liver transplantation

**DOI:** 10.1038/s41598-020-76994-3

**Published:** 2020-11-17

**Authors:** M. Grąt, K. Grąt, M. Krawczyk, Z. Lewandowski, M. Krasnodębski, Ł. Masior, W. Patkowski, K. Zieniewicz

**Affiliations:** 1 Department of General, Transplant and Liver Surgery, Medical University of Warsaw, Warsaw, Poland; 2 Second Department of Clinical Radiology, Medical University of Warsaw, Banacha 1A, 02-097 Warsaw, Poland; 3 Department of Epidemiology and Biostatistics, Medical University of Warsaw, Warsaw, Poland; 4 Second Department of General, Vascular and Oncological Surgery, Medical University of Warsaw, Warsaw, Poland

**Keywords:** Gastrointestinal diseases, Nutrition disorders, Gastroenterology, Hepatology, Medical research, Outcomes research

## Abstract

Perioperative use of probiotics serves as efficient prophylaxis against postoperative infections after liver transplantation, yet data on long-term effects of pre-transplant probiotic intake is lacking. The aim of this study was to assess the effects of pre-transplant probiotic administration on long-term results of liver transplantation. This was secondary analysis of a randomized trial. Patients were randomized to receive either 4-strain probiotic or placebo before liver transplantation. Five year graft survival was set as the primary end-point. Secondary end-points comprised serum bilirubin and C-reactive protein (CRP) concentration, international normalized ratio (INR), serum transaminases and gamma-glutamyl transferase (GGT) activity. Study group comprised 44 patients, of whom 21 received probiotics and 23 received placebo with 5-year graft survival of 81.0% and 87.0%, respectively (*p* = 0.591). Patients in the probiotic arm exhibited lower INR (*p* = 0.001) and CRP (*p* = 0.030) over the first 6 post-transplant months. In the absence of hepatitis B or C virus infection, pre-transplant administration of probiotics also reduced aspartate transaminase activity (*p* = 0.032). In the intervention arm, patients receiving probiotics for under and over 30 days had 5-year graft survival rates of 100% and 66.7%, respectively (*p* = 0.061). Duration of probiotic intake > 30 days was additionally associated with increased INR (*p* = 0.031), GGT (*p* = 0.032) and a tendency towards increased bilirubin (*p* = 0.074) over first 6 post-transplant months. Pre-transplant administration of probiotics has mild positive influence on 6-month allograft function, yet should not exceed 30 days due to potential negative effects on long-term outcomes. (ClinicalTrials.gov Identifier: NCT01735591).

## Introduction

Alterations in gut microbiota are involved in pathogenesis and progression of various chronic liver diseases through a complex system of interactions, commonly referred to as the gut-liver axis^1–3^. The spectrum of diseases at least partially dependent on gut dysbiosis includes non-alcoholic fatty liver disease, alcoholic liver disease, and hepatocellular carcinoma, among other^4–7^. Administration of probiotics is therefore being extensively studied to target the gut dysbiosis in order to improve or slow the progression of various chronic liver diseases.

The positive effects of probiotic intake reported for patients with liver cirrhosis include improvement of liver function, attenuation of portal hypertension, decreased severity of hepatic encephalopathy episodes, attenuation of systemic inflammation, and improvement in immune response^8–10^. In patients with non-alcoholic fatty liver disease, probiotic interventions were associated with improvement of both biochemical and morphological measures of disease severity^11,12^. A recent meta-analysis revealed that in general, administration of probiotics improves liver function tests, particularly in a setting of underlying liver disease, the use of synbiotics, and longer duration of intervention^13^. The underlying mechanisms of action comprise inhibition of lipopolysaccharide/toll-like receptor pathway through decreased endotoxin concentrations, improvement of intestinal vascular barrier, and inhibition of growth of pathogenic species in the intestine lumen^14,15^. Notably, studies on animal models revealed that administration of probiotics ameliorate ischemia–reperfusion injury and improves liver redox status^16,17^. Further, the negative effects of lipopolysaccharide challenge, partially resembling portal reperfusion in liver transplantation, were attenuated by probiotics^18^.

The aforementioned benefits of probiotic intake point towards their potential usefulness in liver transplant setting. Given in the perioperative period, probiotics were proven to decrease the rate of postoperative infections without any remarkable risk of adverse effects^19–24^. Further, a single randomized trial performed in our department revealed that continuous administration of probiotics in the pre-transplant period improves early biochemical parameters of graft function and injury, namely serum bilirubin concentration and transaminases activity^24^. There is, however, no data whether long-term pre-transplant probiotic intake has any effects on post-transplant patient outcomes. While potential protective effects of probiotics may improve liver transplant results by decreasing infection rate and ameliorating ischemia–reperfusion injury, reversion of gut dysbiosis occurs as early as 6 months post-transplantation even without interventions to modulate gut microbiota^25^. The aim of this study was to evaluate the effects of continuous pre-transplant probiotic intake on long-term outcomes of patients after deceased donor liver transplantation.

## Methods

This is a post-hoc analysis of the randomized controlled trial for which data on primary and secondary outcome measures were published previously^24^. Of the 55 patients enrolled in the trial, 44 patients who underwent liver transplantation in the Department of General, Transplant and Liver Surgery in the period between December 2012 and April 2015 were included. The patients were recruited in the period between November 2012 and March 2015. All participants provided informed consent before inclusion in the study. The study was approved by the local ethics committee of the Medical University of Warsaw (KB196/2011). All the methods were in accordance with the Declaration of Helsinki and national regulations. No organs were procured from prisoners. All organs were procured by the transplant team of the Department of General, Transplant and Liver Surgery of the Medical University of Warsaw.

Specific details on recruitment, randomization, blinding, and sample size calculation were provided in the previous paper^24^. The criteria for inclusion in the trial were age of at least 18 years, liver cirrhosis, established underlying liver disease, and inclusion on liver transplant waiting list. Patients were excluded in case of immunosuppressive treatment before transplantation, presence of malignancy, renal function impairment, cystic fibrosis, and human immunodeficiency virus infection.

Participants were randomly allocated with a 1:1 ratio to intervention and control groups based on drawing a sealed envelope containing intervention code performed by the investigators. Randomization was blocked (n = 40) and stratified by Child-Turcotte-Pugh class. Patients in the intervention group received 3 × 10^9^ colony-forming units of *Lactococcus lactis* PB411 (50.0%)*, Lactobacillus casei* PB121 (25.0%)*, Lactobacillus acidophilus* PB111 (12.5%)*,* and *Bifidobacterium bifidum* PB211 (12.5%) in capsules (ProBacti 4 Enteric, Institut Rosell, Canada) daily from inclusion until transplantation. Patients in the control group received placebo in the form of capsules of identical appearance and taste to that administered in the intervention group once daily from inclusion until transplantation. Placebo consisted of bulking and anti-caking agents in the probiotic capsules: potato starch, cellulose, and magnesium stearate. One capsule per day of either placebo or probiotic was administered. Compliance was assessed by collecting empty boxes and by patient interviews. Patients, surgeons, and other care-providers were blinded to randomization results until the end of the original study.

The primary factor of interest was duration of pre-transplant probiotic intake. Patients were categorized into short-term intake group (< 30 days) and long-term intake group (> 30 days). The primary outcome measure for this post-hoc analysis was graft survival, defined as time from transplantation to retransplantation or patient death irrespective of the cause (combined end-point) and censored at the time of last follow-up. Secondary outcome measures included changes of several laboratory measures over 6-month post-transplantation period, including serum bilirubin concentration, activity of aspartate (AST) and alanine (ALT) transaminase, gamma-glutamyl transferase (GGT), international normalized ratio (INR) for prothrombin time, and c-reactive protein (CRP) concentration.

Quantitative variables were presented as medians with interquartile ranges (IQR) or means with 95% confidence intervals (95% CIs). Qualitative variables were presented as numbers with frequencies. Fisher’s exact test and Mann Whitney U test were used for intergroup comparison of baseline characteristics, as appropriate. Kaplan–Meier estimator was used to calculate graft survival and log-rank test was used to evaluate differences between survival curves. Mixed models with repeated measurements were applied to find differences in laboratory values. Effect sizes were calculated as differences of least squares means of mixed models. In the entire cohort analyses, mixed models analyses were adjusted for donor risk index. The level of significance was set to 0.05. All p values were two-sided. Statistical analyses were computed using SAS v. 9.4 (SAS Institute, Cary, NC) and STATISTICA v. 13.1 (Dell Inc., Tulsa, USA).

## Results

The study cohort included 21 patients receiving probiotics (intervention group) and 23 patients receiving placebo (control group) in the pre-transplantation period (Table 1). There were no significant differences between group regarding recipient age (*p* = 0.735), recipient gender (*p* = 0.724), Child-Turcotte-Pugh class (*p* = 0.785), model for end-stage liver disease (*p* = 0.310), hepatitis C virus infection rate (*p* = 0.999), hepatitis B virus infection rate (*p* = 0.481), alcoholic liver disease rate (*p* = 0.521), duration of graft ischemia (*p* = 0.897), donor age (*p* = 0.991), donor risk index (*p* = 0.948), and caval anastomosis technique (*p* = 0.999). However, patients in the probiotics group had significantly lower rate of hepaticojejunostomies (*p* = 0.049). Probiotic intake lasted < 30 and > 30 days in 9 patients (42.9%) and 12 patients (57.1%) in the intervention group, respectively, with the corresponding rates of placebo intake of 30.4% (7 of 23) and 69.6% (16 of 23) in the control group, respectively (*p* = 0.533). Median duration of pre-transplant probiotic and placebo intake was 44 and 41 days, respectively.

**Table 1:** Comparison of baseline characteristics between liver transplant recipients receiving probiotics and those receiving placebo in the pre-transplant period.

Characteristics	Intervention group (n = 21)	Control group (n = 23)	*p*
Duration of intervention: < 30 days	9 (42.9%)	7 (30.4%)	.533
> 30 days	12 (57.1%)	16 (69.6%)	
Male recipient gender	17 (81.0%)	17 (73.9%)	.724
Recipient age (years)	52 (47–58)	50 (35–61)	.735
Child-Turcotte-Pugh class: A	5 (23.8%)	8 (34.8%)	.785
B	11 (52.4%)	11 (47.8%)	
C	5 (23.8%)	4 (17.4%)	
Model for end-stage liver disease	12 (11–18)	13 (10–16)	.310
Hepatitis C virus infection	6 (28.6%)	7 (30.4%)	.999
Hepatitis B virus infection	6 (28.6%)	4 (17.4%)	.481
Alcoholic liver disease	8 (38.1%)	6 (26.1%)	.521
Graft total ischemia (minutes)	559 (452–626)	553 (477–614)	.897
Donor age (years)	51 (41–61)	52 (42–57)	.991
Donor risk index	1.69 (1.42–1.94)	1.76 (1.42–1.86)	.948
Piggyback implantation technique	19 (90.5%)	20 (87.0%)	.999
End-to-end duct-to-duct biliary anastomosis	18 (85.7%)	13 (56.5%)	.049

Data are presented as median (interquartile range) or as n (%).

**Figure 1:**
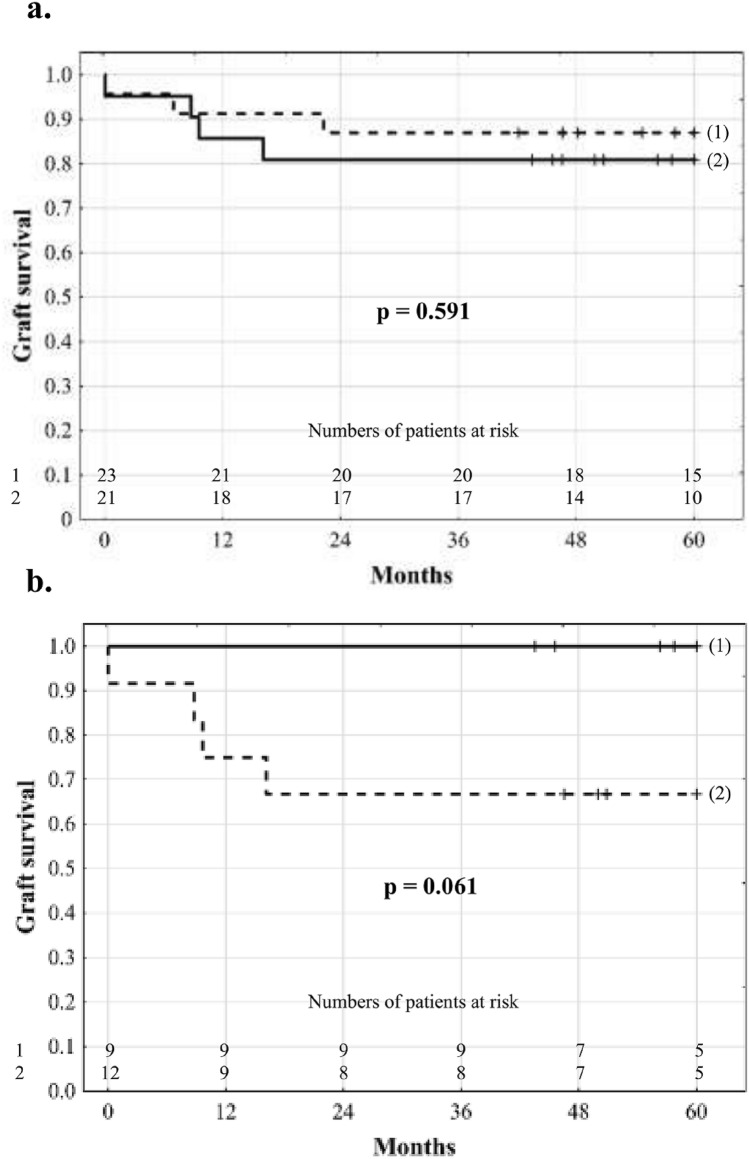
Comparison of 5-year graft survival between (**a**) patients receiving probiotics (solid line) and placebo (dashed line) in the pre-transplant period and (**b**) patients receiving probiotics for under (solid line) and over (dashed line) 30 days.

Median duration of follow-up was 63.4 months. There were 5 deaths. Retransplantations were performed in 3 patients. In the entire study cohort, graft survival rates were 88.6% at 1 year and 84.1% at 3 and 5 years. Five-year graft survival was similar in patients in the intervention (81.0%) and control (87.0%) groups (*p* = 0.591; Fig. 1a). However, 5-year graft survival rate of patients in the intervention group receiving probiotics for > 30 days was 66.7% as compared to 100% of those receiving probiotics for < 30 days (*p* = 0.061; Fig. 1b).

Notably, all of the three patients who died in the subgroup receiving probiotics for > 30 days had hepatitis C virus reinfection, including two with fibrosing cholestatic hepatitis. Both of these two patients died in the course of allograft failure. The cause of death for the third of these patients was neuroinfection and myositis that occurred during therapy with interferon and ribavirin.

**Figure 2:**
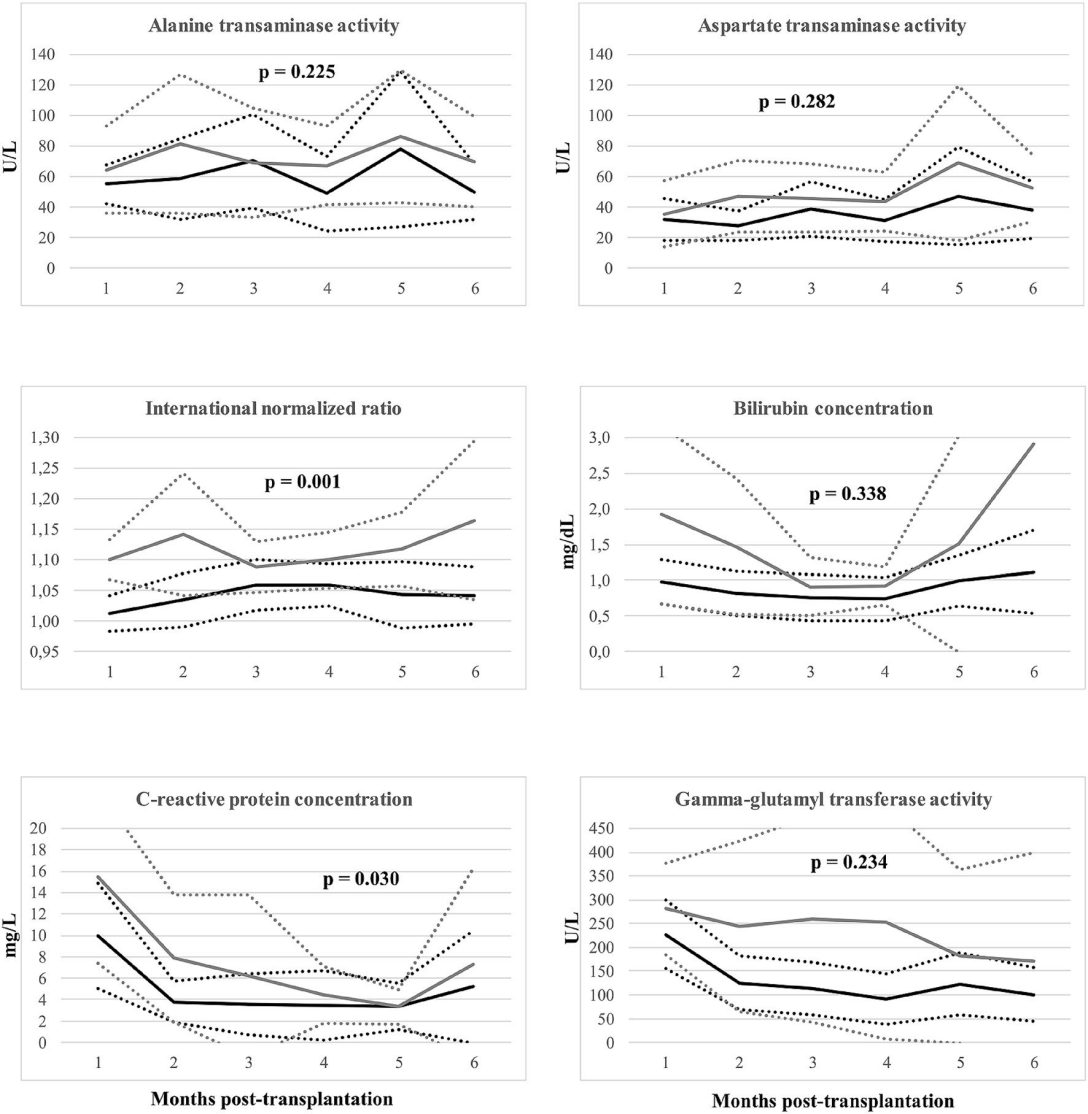
Comparison of selected laboratory parameters over 6-month period after liver transplantation between patients receiving probiotics (black lines) and placebo (grey lines) before the procedure. Solid lines represent means and are presented with 95% confidence intervals (dotted lines).

Patients in the intervention group had significantly lower INR (*p* = 0.001) and CRP concentration (*p* = 0.030) throughout the first 6 month post-transplantation (Fig. 2; Table 2).

**Table 2:** The impact of pre-transplant probiotic intake on selected laboratory parameters over the 6-month period after liver transplantation.

Laboratory measure	All patients Control group, n = 23 Intervention group, n = 21		Patients without HCV or HBV infection Control group, n = 13 Intervention group, n = 12	
	Effect estimate (95% CI)^a^	**p**	Effect estimate (95% CI)^a^	*p*
Aspartate transaminase activity	− 8.5 (− 24.2 to 7.3)	.282	− 7.8 (− 14.8 to − 0.7)	.032
Alanine transaminase activity	− 13.5 (− 35.7 to 8.7)	.225	− 9.1 (− 26.7 to 8.5)	.296
Bilirubin concentration	1. (− 0.1 to 0.2)	.338	0.0 (− 0.4 to 0.4)	.922
International normalized ratio	− 0.05 (− 0.09 to − 0.02)	.001	− 0.08 (− 0.12 to − 0.03)	.001
Gamma-glutamyl transferase activity	− 61.3 (− 163.9 to 41.3)	.234	7.3 (− 118.7 to 133.4)	.905
C-reactive protein concentration	− 1.6 (− 3.1 to − 0.2)	.030	0.5 (− 2.4 to 3.4)	.718

a—intervention vs control group.

HCV—hepatitis C virus; HBV—hepatitis B virus; 95% CI—95% confidence interval.

AST, ALT, GGT and bilirubin were non-significantly lower in patients receiving probiotics as compared to those receiving placebo (Fig. 2).

In a subgroup of patients without hepatis C or B viruses infection, pre-transplant probiotic intake was additionally associated with significantly lower AST (*p* = 0.032). However, patients receiving probiotics for > 30 days before transplantation had higher INR (*p* = 0.031) and GGT activity (*p* = 0.032) and a tendency towards increased bilirubin concentration (*p* = 0.074) over the first 6 post-transplant months than patients receiving probiotics for < 30 days (Table 3).

**Table 3:** The impact of pre-transplant probiotic intake exceeding 30 days on selected laboratory parameters over the 6-month period after liver transplantation.

Laboratory measure	Intervention group Probiotic intake < 30 days, n = 9 Probiotic intake > 30 days, n = 12	
	Effect estimate (95% CI)^a^	*p*
Aspartate transaminase activity	− 11.7 (− 27.9 to 4.5)	.147
Alanine transaminase activity	4.2 (− 7.2 to 15.6)	.448
Bilirubin concentration	0.2 (0.0 to 0.5)	.074
International normalized ratio	0.03 (0.01 to 0.06)	.031
Gamma-glutamyl transferase activity	73.3 (6.9 to 139.7)	.032

a—probiotic intake > 30 days versus under < 30 days.

95% CI—95% confidence interval.

## Discussion

Previously published results on the original primary and secondary outcome measures of this study pointed towards a remarkable reduction in postoperative infection rates and amelioration of ischemia–reperfusion injury with continuous pre-transplant administration of the probiotic regimen^24^. Notably, the intervention seemed to have a favorable safety profile, given no major adverse events related to probiotic intake and similar rates of the reported gastrointestinal symptoms in the probiotic and placebo groups. However, the results of this post-hoc analysis indicate that despite its clinically irrelevant positive effects regarding INR and systemic inflammation, as reflected by CRP concentration, prolonged pre-transplant intake of probiotics may exert negative effects on post-transplant patient outcomes.

Perioperative administration of probiotics in patients undergoing liver transplantation is being increasingly recognized as a preventive measure against postoperative infections^19–24,26^. While one previous randomized trial assessed the effects of continuous pre-transplant probiotic intake on early post-transplant outcomes, the long-term consequences of such intervention remained unknown^24^. Given the protective effects on ischemia–reperfusion injury and modulation of gut microbiota at the time of transplantation, continuous pre-transplant probiotic intake was hypothesized to have positive effects on long-term allograft function, especially in the context of liver function improvement in a population of cirrhotic patients^8^.

No benefits regarding graft survival associated with pre-transplant probiotic administration in general were observed. Despite small numbers, patients receiving probiotics for more than 30 days prior to transplantation had remarkably lower graft survival rate at 5 years than those with probiotic intake not exceeding 30 days, with the difference being on the verge of significance. While there is no clear explanation of this phenomenon, patients with probiotic intake > 30 days additionally had significantly higher INR and GGT as compared to those receiving probiotics for < 30 days, in contrast to the general difference between the intervention and control groups. This may suggest that, unexpectedly, prolonged administration of probiotics before liver transplantation has a negative effect on allograft function. The potential reasons may be related to the modulatory effect on gut-liver axis of the long-term probiotics intervention. Although the present study provided no data on the effects of pre-transplant probiotic administration < 30 days on gut microbiota, 10-week intervention was previously found to be associated with increase in the abundance of *Bacteroides* and *Enterococcus*^24^. Increased *Enterococcus* counts are known to alter liver function in a population of liver transplant candidates^3^. Importantly, all deaths in patients receiving probiotics for more than 30 days occurred in the setting of recurrent hepatitis C virus infection. Gut dysbiosis characterized by increased *Bacteroides* counts and decreased bacterial diversity are found in the course of hepatitis C virus infection^27,28^. Finally, increased *Lactobacillus* and *Bifidobacterium* counts, the bacterial strains included in the regiment, are also known to be increased in the setting of hepatitis C virus-dependent hepatic injury^29^. Accordingly, as the lower diversity of gut microbiota with increased abundance of *Bacteroides*, *Lactobacillus*, and *Bifidobacterium* may be implicated in the pathogenesis of hepatitis C virus infection, administration of the probiotic regimen utilized in the present study may unexpectedly worsen this state of gut dysbiosis at the time of transplantation, yet this hypothesis remains to be elucidated.

The results of this study are insufficient to provide any clear evidence on casuality between prolonged intake of probiotics and inferior graft survival. However, given the patient numbers, the difference in graft survival rates on the verge of statistical significance cannot be omitted and should be considered as an argument for cautious administration of probiotics before liver transplantation. The positive effects of probiotic intake before liver transplantation with respect to lower INR values over the first 6 months after transplantation are unlikely to reflect improved allograft function, given the effect size not exceeding 0.1. However, the difference with respect to INR may indirectly reflect changes of gut microbiota composition related to pre-transplant probiotics, as vitamin K metabolism is influenced by intestinal bacteria^30^. Accordingly, increased INR in patients receiving probiotics for more than 30 days may point towards the negative effects of prolonged administration on gut dysbiosis, in line with increased *Bacteroides* abundance reported previously^24^. Notably, increased abundance of *Bacteroides* and associated enhanced immune response was previously reported as one of benefits of probiotic administration^31^. In the setting of liver disease, this in fact may be a negative consequence of probiotic intake as illustrated by a recent study indicating aggravation of pro-inflammatory response related to higher abundance of these bacteria in the gut^32^. Nevertheless, probiotic intake in general was associated with lower CRP concentrations in all patients and with lower AST activity in patients without hepatitis B or C virus infection, which points towards its mild protective effect on the allograft. This is in line with the results of previous studies indicating improvement in liver function tests and reduced systemic inflammation associated with administration of probiotics^12,13^. The observed differences yet do not appear to be clinically relevant.

The present study has several limitations. First, this is a post-hoc analysis utilizing relatively small numbers of patients. Second, there were no data on post-transplant changes in the composition of gut microbiota, which limits the ability to explain the potential reasons for negative consequences of prolonged probiotic intake. Further, the duration of pre-transplant probiotic administration was not random, as it was directly related to the waiting time. However, the selection bias should influence the results in the opposite fashion, as patients with shorter waiting time are expected to be in worse condition and have higher clinical urgency for transplantation. Finally, the results of this post-hoc analysis remain limited to the probiotic regimen containing 3 × 10^9^ colony-forming units of *Lactococcus lactis* PB411 (50.0%)*, Lactobacillus casei* PB121 (25.0%)*, Lactobacillus acidophilus* PB111 (12.5%)*,* and *Bifidobacterium bifidum* PB211 (12.5%).

In conclusion, the results of the present study indicate that probiotic intake for more than 30 days before liver transplantation may exert negative effects on post-transplant outcomes. This may be considered as a safety alert, especially for patients undergoing transplantations for hepatitis C virus-related cirrhosis.

## Data Availability

The data used for this study are available from the authors upon a reasonable request.
